# Impulse Control Disorder in Parkinson's Disease: A Meta-Analysis of Cognitive, Affective, and Motivational Correlates

**DOI:** 10.3389/fneur.2018.00654

**Published:** 2018-08-28

**Authors:** Alice Martini, Denise Dal Lago, Nicola M. J. Edelstyn, James A. Grange, Stefano Tamburin

**Affiliations:** ^1^School of Psychology, Keele University, Newcastle-under-Lyme, United Kingdom; ^2^Department of Neurosciences, Biomedicine and Movement Sciences, University of Verona, Verona, Italy

**Keywords:** Parkinson's disease, impulse control disorder, cognition, affective factors, motivation, impulsivity, meta-analysis, depression

## Abstract

**Background:** In Parkinson's disease (PD), impulse control disorders (ICDs) develop as side-effect of dopaminergic replacement therapy (DRT). Cognitive, affective, and motivational correlates of ICD in medicated PD patients are debated. Here, we systematically reviewed and meta-analyzed the evidence for an association between ICD in PD and cognitive, affective, and motivational abnormalities.

**Methods:** A systematic review and meta-analysis was performed on PubMed, Science Direct, ISI Web of Science, Cochrane, EBSCO for studies published between 1-1-2000 and 8-3-2017 comparing cognitive, affective, and motivational measures in PD patients with ICD (ICD+) vs. those without ICD (ICD–). Exclusion criteria were conditions other than PD, substance and/or alcohol abuse, dementia, drug naïve patients, cognition assessed by self-report tools. Standardized mean difference (SMD) was used, and random-effect model applied.

**Results:** 10,200 studies were screened (title, abstract), 79 full-texts were assessed, and 25 were included (ICD+: 625 patients; ICD–: 938). Compared to ICD–, ICD+ showed worse performance reward-related decision-making (0.42 [0.02, 0.82], *p* = 0.04) and set-shifting tasks (SMD = −0.49 [95% CI −0.78, −0.21], *p* = 0.0008). ICD in PD was also related to higher self-reported rate of depression (0.35 [0.16, 0.54], *p* = 0.0004), anxiety (0.43 [0.18, 0.68], *p* = 0.0007), anhedonia (0.26 [0.01, 0.50], *p* = 0.04), and impulsivity (0.79 [0.50, 1.09], *p* < 0.00001). Heterogeneity was low to moderate, except for depression (*I*^2^ = 61%) and anxiety (*I*^2^ = 58%).

**Conclusions:** ICD in PD is associated with worse set-shifting and reward-related decision-making, and increased depression, anxiety, anhedonia, and impulsivity. This is an important area for further studies as ICDs have negative impact on the quality of life of patients and their caregivers.

## Introduction

Impulse control disorders (ICDs), such as pathological gambling, hypersexuality, binge-eating, and compulsive shopping, can occur in over 13% of medicated Parkinson's disease (PD) patients (1). Although ICDs are recognized as side-effect of dopamine replacement therapy (DRT), mainly D2 dopamine agonists and levodopa, their pathophysiology is unclear.

It has been hypothesized that, in vulnerable individuals, DRT used to restore dopamine levels in nigrostriatal circuitry may overstimulate the less severely affected mesocorticolimbic circuitry (2). Mesocorticolimbic overstimulation may disrupt prefrontal-dependent executive function, affect and motivation and thus increase vulnerability to ICD. According to this view, in medicated PD patients, we should expect a correlation between ICD and cognitive, affective and motivational factors. However, data in the literature are inconclusive.

Studies on cognition, affective processing and motivation conducted in small cohorts of PD patients with and without ICD (i.e., n: 17–155 patients) yielded inconsistent findings with respect to frontal cognitive abilities in PD patients with ICD. Some studies reported worse performance in executive function, including set-shifting (3–7), working memory (8), concept formation and reasoning (5, 7), and reward-related decision-making (9–15) in PD with ICD (ICD+) compared to PD without ICD (ICD-). Conversely, other studies found similar performances for inhibition (9, 16–18), set-shifting (19, 20), working memory (3, 11, 17, 21, 22), and reward-related decision-making (16, 17, 20, 23). Finally, a single study reported better executive functions in ICD+ (24). Reports on affective factors are also inconclusive, as self-reported depression and anxiety were sometimes found to be associated with ICD (18, 20, 21, 25–28), and sometimes not (3–6, 17, 19, 22, 29–31). However, motivational factors such as self-reported apathy (11, 21, 27, 28), anhedonia (27, 32), and impulsivity (17, 20–22, 32) appeared to be elevated in ICD+ vs. ICD–.

A recent meta-analysis identified several cognitive subdomains (i.e., concept formation, set-shifting, reward-related decision-making, and visuospatial abilities) to be worse in ICD+ vs. ICD– (33), but it included a mixed sample of medicated and drug naïve patients that did not allow to explore the relationship between cognitive disturbances, DRT and ICD.

Moreover, it included patients with comorbidities for substance abuse and/or dementia, two factors that could be independently associated with cognitive changes. Finally, the relationship between cognition-emotion and cognition-motivation, critical to understanding the broader context in which ICDs develop, was not explored in the previous meta-analysis (34).

To reconcile discordant findings in the literature about cognitive, affective and motivational correlates of ICD in medicated PD patients, a systematic review and meta-analysis was conducted. Moreover, this work is meant to address the issues of a previous meta-analysis and to offer new information on this topic. To this aim, we applied stricter inclusion and exclusion criteria, by including only studies on PD patients under DRT at the time of assessment and free from co-morbid substance abuse and/or dementia. Moreover, we included studies with affective and motivational measures, so that any cognitive change could be interpreted within the broader context of cognition-emotion and cognition-motivation relationships (34). A clear understanding of cognitive, affective and motivational changes in ICD may indirectly increase our understanding of ICD pathophysiology and in turn its management.

## Methods

### Study design, participants, and comparators

A systematic review and meta-analysis were performed to identify cognitive, affective and motivational factors associated with ICD in PD under DRT (ICD+). The comparator group was patients with PD but no ICD (ICD–).

### Search strategy and selection criteria

On June 26th 2016, PubMed, Science Direct, ISI Web of Science, Cochrane, EBSCO were searched for peer-reviewed papers in English, Italian and Spanish published since January 2000, when the first report of ICD development after dopaminergic medication initiation was reported (35). The systematic review was further updated on March 8th 2017.

Studies were identified using the following string (36) in PubMed: “(Parkinson's disease) AND (impulse control disorders OR impulsivity OR cognition OR decision-making).” The search strategy for the other databases included (Parkinson's disease) AND (impulse control disorders), then (Parkinson's disease) AND (impulsivity), then (Parkinson's disease) AND (cognition), and (Parkinson's disease) AND (decision-making). A total of 40,672 papers were identified. After exclusion of duplicates, 10,200 papers were title and abstract screened.

Studies were included if: (a) PD patients were under DRT; (b) ICD assessment was performed in a reliable manner with the Questionnaire for Impulsive-Compulsive Disorders in Parkinson's Disease (QUIP), the QUIP rating scale (QUIP-rs), the Minnesota Impulse Disorders Interview, clinical interview based on diagnostic criteria, or a combination of these; (c) performances of PD patients with ICD (ICD+) were compared with those with PD but no history of ICD (ICD–); (d) cognitive, affective, and/or motivational measures were reported. A further inclusion criterion was independence of samples. Only baseline data for prospective studies and the study with the largest sample for multiple studies published by the same author(s) were included.

We excluded reviews, case studies, commentaries, letters, abstracts and dissertations, and postal surveys. Studies including drug naïve PD patients were also excluded since we were interested in ICD developed as a DRT side-effect. Studies in which PD patients underwent non-pharmacological treatments such as deep brain stimulation (DBS) were excluded. This criterion was based on controversial reports of either ICD amelioration or ICD appearance after DBS (37), and the notion that DBS may worsen some cognitive outcomes (38). Studies including participants with dementia and drug/alcohol abuse were excluded, as these conditions might be independently associated with cognitive and neuropsychiatric changes. Other exclusion criteria were: cognition assessed by self-report measures or by general screening tools (e.g., Mini-Mental State Examination) because of their limited specificity and sensitivity (39). Studies focusing on dopamine dysregulation syndrome and/or punding only were not included since these conditions are considered different from ICD, as they are more common in patients with advanced PD, cognitive impairment and dementia (40). However, screening questionnaires (e.g., QUIP, QUIP-rs) include dopamine dysregulation syndrome and punding, and some ICD+ patients we included may have had these conditions too, in addition to ICD. Finally, to ensure that the ICD- group included patients without any type of ICD, studies not assessing all ICD types (e.g., using only the South Oaks Gambling Screen) were excluded.

### Data extraction

Following exclusion of duplicate and irrelevant articles through title and abstract screening, 79 papers were included for full-text evaluation. Reference lists of these studies were manually searched to identify additional relevant articles, and two papers were included at this stage.

**Figure 1:**
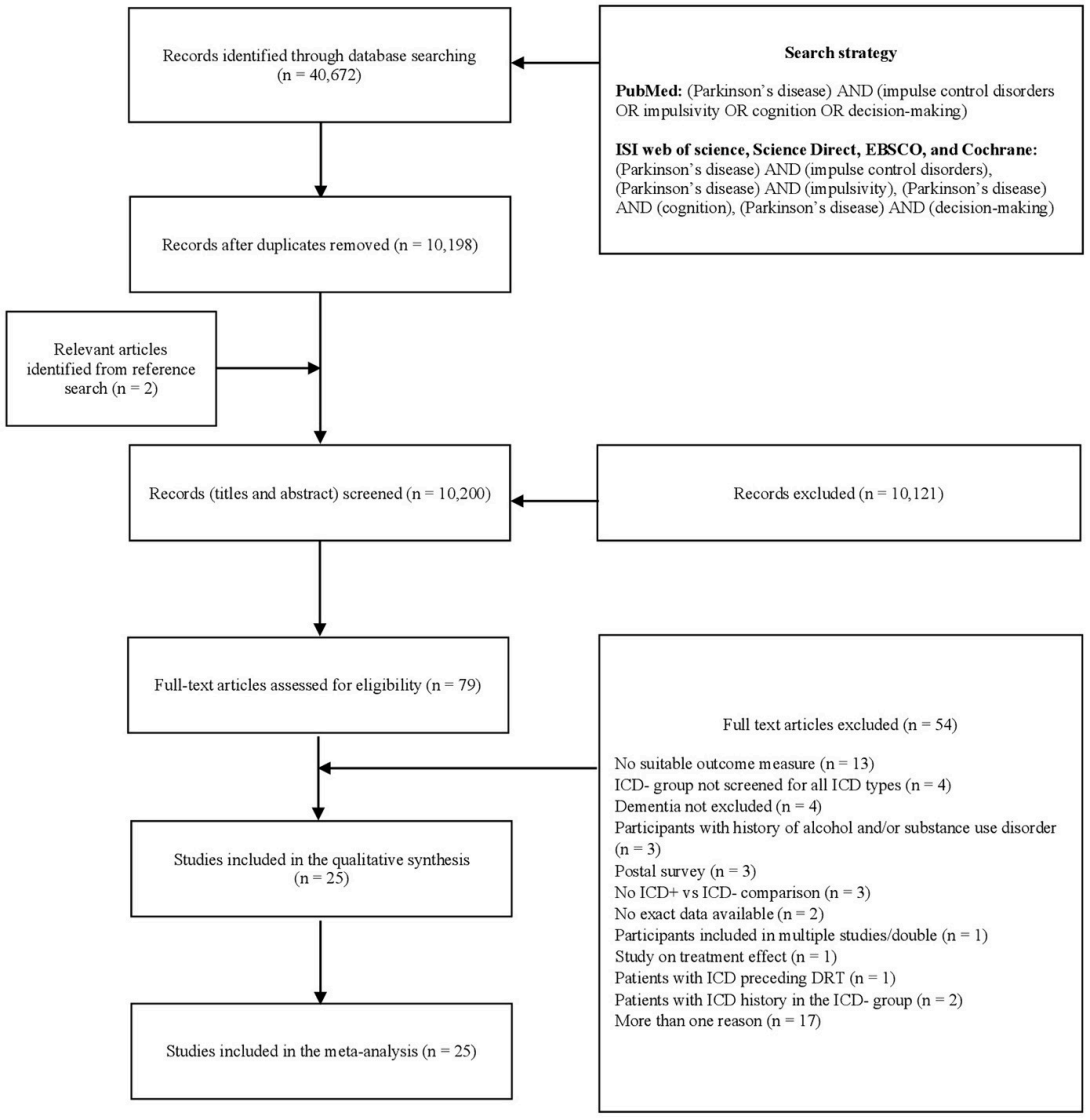
PRISMA diagram of the study (www.prisma-statement.org). DRT, dopaminergic replacement treatment; ICD, impulse control disorder; ICD+, PD patients with ICD; ICD–, PD patients without ICD; PD, Parkinson's disease.

Two reviewers (AM, DDL) independently screened titles and abstracts using Rayyan software (41), and three reviewers (AM, DDL, ST) independently evaluated papers selected for full-text examination. Disagreements were resolved through discussions. Disagreement concerned one paper (42) over the 75 selected for full-text examination (inter-rater agreement: 98.67%). Twenty-five articles were included for quantitative analysis (Figure 1).

Corresponding authors of five studies were contacted for exact data. Means and standard deviations were obtained for two studies, which reported median and interquartile ranges (20, 25), according to a proposed formula (43). Two reviewers (AM, DDL) independently extracted the following data: sample size, age at evaluation, age at PD onset, PD duration, education (years), Hoehn and Yahr (H and Y) stage, Unified Parkinson's Disease Rating Scale motor section (UPDRS-III) ON-medication, depression, antidepressants use, antipsychotics use, total levodopa equivalent daily dose (LEDD, mg), levodopa LEDD, dopamine agonist LEDD, outcomes, ICD screening tool, ICD type, and statistics.

Primary outcomes were cognitive, affective, and motivational scores. Cognitive tests were categorized on the basis of the main cognitive process involved (44). The categories were “memory”(short-term verbal and visuospatial memory, long-term verbal and visuospatial memory); “working memory”; “attention”; “executive function” (concept formation and reasoning, concept formation sort and shift, set-shifting, inhibition, cognitive flexibility, reward-related decision-making); “visuospatial abilities”; “language”; “apraxia”; “novelty seeking”; “incentive salience” and “data gathering.” Concept formation and reasoning relates to the development of ideas based on the common properties of objects, events, or qualities using abstraction and generalization processes whilst concept formation sort and shift requires to form a sorting principles and apply it (sort), and then abandon it and switch to a different principle (shift) (44).

Affective and motivational measures were categorized as depression, anxiety, anhedonia, apathy, and impulsivity.

Cognitive processes assessed in a single study (i.e., novelty seeking, incentive salience, data gathering, apraxia) were not included in the meta-analysis. When a study reported multiple measures for the same outcome, the most relevant one was chosen by two reviewers with expertise on neuropsychological assessment (AM, DDL).

### Data analysis

Data were analyzed using ReviewManager v5.3 (45). Effect size was estimated as standardized mean difference (SMD), which is comparable to Hedges' adjusted g value. Effect sizes of 0.2, 0.5, and 0.8 or more are considered as small, moderate and large, respectively (46). Cochran's Q (χ^2^) was used to test heterogeneity between studies. The degree of heterogeneity was quantified by *I*^2^, which values range between 0 and 100%. *I*^2^ percentages of 25, 50, 75 are considered as low, moderate and high, respectively (47). Random-effect model was applied, as patients differ in clinical (e.g., UPDRS-III ON medication range: 10.9–36.7) and demographic characteristics (e.g., age range: 54.6–71.4), therefore the true effect may vary from study to study. In contrast to fixed-effect models, random-effect models consider both within and between study variances. As heterogeneity was moderate to high for some outcomes (i.e., working memory, depression, anxiety, and apathy), the consequences of applying a fixed-effect model, which does not consider between studies variance, may result in type I error rate inflation (48). Conversely, if random-effect models are applied with effect sizes that vary only due to sampling error as when heterogeneity is low (i.e., short-term visuospatial memory, attention, concept formation reasoning, anhedonia), the consequences are less dramatic (e.g., using Hedges' method, the additional between-study effect size variance used in the random effect method becomes zero when sample effect sizes are homogeneous, yielding the same result as the fixed effect method) (48). Moreover, following this approach, studies were not excluded because of their small sample size, because in random-effect models effect sizes are weighed by their variance, which is higher in smaller studies.

Two authors independently explored funnel plots for publication bias (AM, DDL), and incongruences were resolved by discussion with two other authors (ST, JAG). Funnel plots of outcomes with less than ten studies were not inspected since the power is too low to discriminate publication bias's asymmetry from chance (49). Blinding of assessors (performance bias) and incomplete data outcome (attrition bias) were independently assessed for each study as “low risk,” “high risk,” or “unclear” by two reviewers (AM, DDL) following Cochrane Collaboration recommendations. Sensitivity analysis was performed by excluding one study at time and verifying its impact on the overall effect size. Sensitivity analysis was not performed for outcomes with two studies. Moderator analysis via meta-regression was performed using SPSS version 21.0 (50). We tested the hypothesis that variation among studies in effect size was associated with differences in age, years of education, disease duration, UPDRS-III score, H and Y score, total LEDD, levodopa LEDD, and dopamine agonist LEDD. As suggested by Borenstein (51), moderator analysis was conducted only for outcomes in which there were at least 10 studies to one covariate.

## Results

After removal of duplicates, 10,200 records were screened by title and abstract, 79 full-text articles were assessed for eligibility, and 54 were excluded (Figure 1). Twenty-five studies were included in the meta-analysis (Table 1).

Table 1Characteristics of the studies included in the meta-analysis.

**Table:** 

**Ref**	**Pts (males)**	Age (y)^*^	PD onset (y)^*^	PD duration (y)^*^	Education (y)^*^	**H and Y**	UPDRS-III (ON)^*^	**Depression^†^**	**Antidepressant (N)**
Bentivoglio et al. (17)	ICD+: 17 (14) ICD–: 17 (11)	ICD+: 62.0 (10.1) ICD–: 63.9 (9.2)	NR	ICD+: 6.9 (3.8) ICD–: 7.3 (4.4)	ICD+: 8.7 (3.7) ICD–: 10.2 (4.4)	ICD+: 2.0 (0.8) ICD–: 2.3 (0.5)	ICD+: 23.8 (11.0) ICD–: 22.5 (6.9)	NO	ICD+: 2 ICD–: 4
Biundo et al. (3)	ICD+: 33 (18) ICD–: 24 (17)	ICD+: 61.3 (10.2) ICD–: 70.4 (6.8)	ICD+: 53.2 (10.6) ICD–: 60.5 (10.0)	ICD+: 8.8 (4.8) ICD–: 8.9 (5.4)	ICD+: 11.8 (3.9) ICD–: 10.4 (4.8)	NR	ICD+: 30.2 (13.2) ICD–: 32.3 (12.8)	NO	NR
Biundo et al. (4)	ICD+:58 (38) ICD–:52 (32)	ICD+: 60.3 (9.3) ICD–: 63.1 (10.2)	ICD+: 50.1 (12.1) ICD–: 54.7 (11.6)	ICD+: 9.0 (5.5) ICD–: 8.0 (5.7)	ICD+: 10.9 (4.3) ICD–: 11.3 (4.7)	ICD+: 2.4 (0.7) ICD–: 2.3 (0.7)	ICD+: 26.7 (16.5) ICD–: 28.5 (12.3)	NO	NR
Cera et al. (16)	ICD+:9 (6) PG:10 (7) ICD–:14 (7)	ICD+: 59.3 (6.8) PG: 60.6 (6.8) ICD–: 59.0 (9.5)	NR	ICD+: 29.0 (8.5)^‡^ PG: 28.2 (12.3) ICD–: 27.2 (8.4)	ICD+: 10.3 (3.2) PG: 11.7 (2.6) ICD–: 11.7(1.9)	ICD+: 1.7 (0.3) PG: 1.9 (0.2) ICD–: 1.7 (0.0)	ICD+: 21.4 (4.2) PG: 20.5 (6.8) ICD–: 21.6 (6.9)	NO	NR
Cilia et al. (30)	ICD+: 11 (10) ICD–: 40 (27)	ICD+: 57.4 (5.8) ICD–: 55 (7)	ICD+: 49.5 (4.7) ICD–: 46.4 (7.2)	ICD+: 8.4 (3.4) ICD–: 8.4 (5.1)	NR NR	ICD+: 2.1 (0.6) ICD–: 2.3 (0.8)	ICD+: 18.0 (11.0) ICD–: 19.1 (8.5)	YES	NO
Claassen et al. (31)	ICD+: 12 (8) ICD–:12 (6)	ICD+: 59.4 (5.5) ICD–: 60.8 (7.2)	NR	ICD+: 6.5 (4.7) ICD–: 6.1 (3.8)	ICD+: 17.1 (2.7) ICD–: 16.3 (2.8)	NR	ICD+: 15.9 (6.6) ICD–: 15.7 (8.3)	YES	NO
Djamshidian et al. (8)	ICD+:18 (13) ICD–:12 (9)	ICD+: 55 (2.1) ICD–: 63.6 (2.2)	ICD+: 43.9 (2.1) ICD–: 50.9 (2.2)	ICD+: 10.9 (1.2) ICD–: 12.7 (2.1)	ICD+: 12.2 (0.9) ICD–: 14.2 (1.3)	NR	ICD+: 18.0 (2.2)^§^ ICD–: 13.0 (1.4)	NO	NR
Djamshidian et al. (9)	ICD+: 28 (21) ICD–:24 (21)	ICD+: 54.6 (9.2) ICD–: 64.2 (10.1)	ICD+: 44.5 (8.7) ICD–: 52.5 (9.6)	ICD+: 10.1 (5.5) ICD–: 11.7 (7.2)	ICD+: 13.4 (3.0) ICD–: 14.7 (3.6)	NR	ICD+: 15.5 (8.3) ICD–: 14.4 (5.8)	NO	ICD+: 4 ICD–: 2
Erga et al. (18)	ICD+: 38 (26) ICD–:87 (49)	ICD+: 67.9 (7.7) ICD–: 71.4 (9.8)	NR	ICD+: 7.4 (1.6) ICD–: 7.4 (1.9)	NR	ICD+: 2.2 (0.5) ICD–: 2.2 (0.6)	ICD+: 23.8 (10.5) ICD–: 22.2 (10.7)	NO	ICD+: 5 ICD–:11
Housden et al. (11)	ICD+: 18 (11) ICD–:18 (12)	ICD+: 62.3 (7.6) ICD–: 67.7 (5.5)	NR	ICD+: 13.9 (9.0) ICD–: 12.9 (8.3)	NR	ICD+: 2.5 (0.6) ICD–: 2.5 (0.7)	ICD+: 20.0 (6.6) ICD–: 21.3 (10.4)	YES	NR
Joutsa et al. (23)	ICD+:9 (9) ICD–:8 (8)	ICD+: 59.3 (8.4) ICD–: 60.1 (5.9)	ICD+: 53.1 (8.7) ICD–: 55.3 (5.1)	ICD+: 6.1 (1.8) ICD–: 5.1 (2.0)	NR	NR	ICD+: 31.7 (4.9) ICD–: 30.1 (10.7)	YES	NR
Leroi et al. (21)	ICD+: 35 ICD–:38	NR	NR	NR	NR	NR	ICD+: 26.9 (10.0) ICD–: 24.1 (10.4)	NO	NR
Mack et al. (19)	ICD+: 17 (11) ICD–:17 (8)	ICD+: 61.1 (7.5) ICD–: 63.8 (8.5)	ICD+: 48.1 (5.2) ICD–: 53.7 (10.0)	ICD+: 13.1 (6.9) ICD–: 10.2 (5.6)	NR	ICD+: 2.8 (1.0) ICD–: 2.4 (1.3)	ICD+: 36.7 (16.1) ICD–: 28.5 (15.2)	NO	YES
Merola et al. (42)	ICD+: 8 (8) ICD–: 113 (60)	NR	ICD+: 48.2 (9.4) ICD–: 46.6 (7.3)	ICD+: 13.4 (7.8) ICD–: 13.1 (4.4)	NR	NR	ICD+: 14.3 (6.7) ICD–: 15.5 (7.8)	NO	NR
O'Sullivan et al. (29)	ICD+:39 (31) ICD–:61 (44)	ICD+: 59.3 (9.1) ICD–: 66.6 (9.5)	ICD+: 45.8 (10.3) ICD–: 55.9 (11.7)	ICD+: 12.0 (6.0) ICD–: 9.6 (7.1)	NR	ICD+: 2.6 (0.5) ICD–: 2.2 (0.5)	ICD+: 16.3 (7.5) ICD–: 18.5 (8.8)	NO	NR
O'Sullivan et al. (28)	ICD+: 30 (26) ICD–: 62 (46)	ICD+: 58.9 (8.5) ICD–: 66.4 (9.7)	ICD+: 46.2 (10.1) ICD–: 55.8 (12.0)	ICD+: 11.5 (5.9) ICD–: 9.5 (7.0)	NR	ICD+: 3 (2–3)^¶^ ICD–: 2 (2–3)	NR	NO	YES
Pettorruso et al. (32)	PG: 11 (8) ICD+: 23 (18) ICD–: 120 (60)	PG: 64.9 (10.9) ICD+: 62.0 (9.1) ICD–: 67.7 (9.4)	PG: 56.6 (10.6) ICD+: 53.2 (9) ICD–: 60.6 (9.2)	PG: 8.3 (3.2) ICD+: 8.8 (6) ICD–: 7.0 (5.4)	PG: 10 (4.2) ICD+: 11.3 (4.4) ICD–: 11 (5.2)	NR	PG: 20.4 (12.3) ICD+: 18.4 (8.5) ICD–: 20.4 (8.4)	NO	NR
Pineau et al. (20)	ICD+: 17 (14) ICD–: 20 (13)	ICD+: 55 (37–69)^||^ ICD–: 55 (40–62)	ICD+: 48 (32–65)^||^ ICD–: 48 (35–55)	ICD+: 7 (2–10)^||^ ICD–: 5.5 (4–12)	ICD+: 7 (3–7)^||^ ICD–: 7 (3–7)	NR	ICD+: 7 (0–23)^||^ ICD–: 8.5 (0–34)	NO	NR
Piray et al. (22)	ICD+: 16 (14) ICD–: 15 (12)	ICD+: 64.4 (3.3) ICD–: 63.3 (4.0)	NR	ICD+: 9.6 (2.5) ICD–: 8.9 (3.1)	NR	ICD+: 2.5 (0.5) ICD–: 2.4 (0.6)	ICD+: 19.0 (5.3) ICD–: 19.6 (6.4)	NO	NR
Pontieri et al. (27)	PG: 21 ICD+: 36 ICD–: 98	PG: 58 (9) ICD+: 64 (8) ICD–: 66 (9)	PG: 51 (8) ICD+: 57 (10) ICD–: 61 (9)	PG: 8 (5) ICD+: 7 (4) ICD–: 5 (3)	PG: 10 (4) ICD+: 11 (4) ICD–: 10 (4)	PG: 2.0 (0.5) ICD+: 1.9 (0.8) ICD–: 1.8 (0.5)	PG: 21.5 (11.6) ICD+: 19.1 (12.7) ICD–: 19.0 (11.9)	NO	PG: 4 ICD+: 7 ICD–: 26
Rossi et al. (10)	ICD+: 7 (6) ICD–: 13 (10)	ICD+: 61.4 (6.9) ICD–: 65.1 (3.8)	ICD+: 52.0 (5.6) ICD–: 58.3 (6.9)	NR	ICD+: 13.8 (4.1) ICD–: 11.9 (5.5)	ICD+: 2.2 (0.7) ICD–: 2.0 (0.7)	ICD+: 17.0 (9.1) ICD–: 14.7 (6.7)	NO	NR
Tessitore et al. (5)	ICD+: 15 (13) ICD–: 15 (12)	ICD+: 62.9 (8.6) ICD–: 63.1 (8.0)	NR	ICD+: 5.3 (2.9) ICD–: 6.6 (3.9)	ICD+: 9.8 (5) ICD–: 12.9 (8)	ICD+: 1.3 (0.5) ICD–: 1.4 (0.6)	ICD+: 10.9 (4.5) ICD–: 12.1 (4.4)	NO	NO
Vela et al. (25)	ICD+: 49 (28) ICD–: 35 (23)	ICD+: 48 (44–52)^¶^ ICD–: 46 (42–52)	NR	ICD+: 7 (3–11)^¶^ ICD–: 3 (1–10)	NR	ICD+: 2 (2–2)^¶^ ICD–: 2 (1–2)	ICD+: 16(10–22)^¶^ ICD–: 17 (11–24)	NO	NO
Vitale et al. (6)	HS: 13 (13) M-ICD: 10 (9) ICD–: 14	HS: 68.7 (5.4) M-ICD: 62.2 (7.5) ICD–: 61.3 (8.2)	HS: 59.5 (5.6) M-ICD: 55.5 (5.3) ICD–: 53.2 (9.1)	HS: 8.5 (3.9) M-ICD: 8.1 (4.5) ICD–: 7.6 (4.4)	HS: 9.5 (5) M-ICD: 8.2 (2.8) ICD–: 13 (4)	HS: 1.8 (0.5) M-ICD: 1.5 (0.7) ICD–: 1.8 (0.8)	HS: 15.1 (6.5) M-ICD: 13 (7.1) ICD–: 11.7 (6)	NO	HS: 1 M-ICD: 2 ICD–: 0
Wu et al. (26)	S-ICD: 7 M-ICD: 10 ICD–: 9	S-ICD: 62.3 (3.9) M-ICD: 58.1 (2.8) ICD–: 60.2 (3.2)	S-ICD: 51.7 (4.0) M-ICD: 43.8 (3.4) ICD–: 50.3 (3.4)	S-ICD: 10.6 (2.0) M-ICD: 14.3 (11.2) ICD–: 9.9 (2.1)	NR	NR	NR	NO	NR

**Table:** 

**Ref**	**Antipsychotic: N**	**LEDD (mg)**			**Outcomes**	**ICD**	
		Total LEDD^*^	LD-LEDD^*^	DA-LEDD^*^		Diagnosis^**^	**Type: N**
Bentivoglio et al. (17)	ICD+: 3	ICD+: 606.1 (319.2) ICD–: 616.2 (367.8)	ICD+: 539 (264.3) ICD–: 455.7 (299.0)	ICD+: 172.9 (112.2) ICD–: 192.5 (88.5)	Digit span forward; CBTT; Immediate visual memory; RAVLT; Digit span backward; Double barrage; FAB; MWCST; RCPM; Stroop; Fluency (semantic, phonological); IGT; Apraxia (ideomotor, orofacial, constructional); Oral confrontation naming (nouns, verbs); HAM-D; HAM-A; BIS-11	Clinical interview (DSM-IV)	HS: 8; CS: 2; PG: 10; BE: 6; M-ICD: 7
Biundo et al. (3)	NR	ICD+: 556.8 (304.6) ICD–: 497.4 (341.2)	NR	ICD+: 186.5 (149.3) ICD–: 165.8 (108.8)	Digit span forward; CBTT; RAVLT; ROCF (copy, delayed); Digit span backward; TMT A; FAB; TMT B; RCPM; Similarities for abstract verbal reasoning; Stroop; Fluency (semantic, phonological); BDI	MIDI; DSM-IV-TR; interview (caregivers); additional clinical interview	HS: 11; CS: 9; PG: 1; punding: 2; M-ICD: 12
Biundo et al. (4)	NR	ICD+: 923.1 (474.1) ICD–: 722.6 (498.5)	NR	ICD+: 163.7 (111.3) ICD–: 148.9 (105.0)	Digit span forward; CBTT; Prose (immediate, delayed); ROCF; Digit ordering test; TMT-A; TMT B; Stroop; Fluency (semantic, phonological); Naming; VOSP; Clock drawing test; BDI	QUIP-RS; MIDI; clinical interview (patient and carergiver)	HS: 6; CS: 7; PG: 2; hoarding: 2; impulsive aggression: 1; M-ICD: 40
Cera et al. (16)	NO	ICD+: 283.3 (132.9) PG: 294.5 (123.1) ICD–: 307 (96.3)	NR	NR	Stroop test; Emotional Stroop test; Monetary risk tasking task	DSM-IV, QUIP-RS, SOGS	PG:10; M-ICD: 9
Cilia et al. (30)	NO	ICD+: 811.8 (229.0) ICD–: 877.3 (289.3)	NR	ICD+: 289.1 (57.5) ICD–: 340.1 (157.2)	FAB; RPM; GDS	Diagnostic criteria; SOGS	PG:1; PG+HS: 5; PG+BE: 2; PG+CS: 2; PG+IA: 1
Claassen et al. (31)	NO	ICD+: 618.7 (361.9) ICD–: 520.3 (314.9)	ICD+: 408.2 (349.6) ICD–: 319.7 (318.9)	ICD+: 293.8 (167.4) ICD–: 200.6 (116.8)	Stop signal task; CESD	QUIP; clinical interview	HS: 5; CS: 5; BE: 6; hobbyism: 9
Djamshidian et al. (8)	NR	ICD+: 971 (183)^§^ ICD–: 732 (203)	ICD+: 752 (109)^§^ ICD–: 604 (73)	NR	Digit span backward; Risk Task; Learning task.	Diagnostic criteria	PG: 10; HS:9; CS: 5; BE: 7; DDS: 6; punding: 2; kleptomania: 1
Djamshidian et al. (9)	NR	ICD+: 832 (425) ICD–: 821 (400)	NR	NR	Stroop	Diagnostic criteria	PG: 11; HS: 13; CS: 8; punding:4; kleptomania:1
Erga et al. (18)	NR	ICD+: 730.6 (343.3) ICD–: 658.4 (275.9)	ICD+: 505.2 (279.1) ICD–: 408.7 (266.7)	ICD+: 293.7 (132.4) ICD–: 289.5 (150.0)	CLVT-II; Stroop; Fluency (phonological); VOSP; MADRS	QUIP	M-ICD: 36 (PG: 2; HS: 7; CS:6; BE:14; punding:12; hobbyism:13; DDS: 3)
Housden et al. (11)	NR	ICD+: 891.5 (432.1) ICD–: 804.8 (358.5)	ICD+: 643.5 (254.1) ICD–: 634.2 (301.7)	ICD+: 248 (301.3) ICD–: 170.5 (159.3)	Digit span forward; Digit span backward; KDDT; WTAR; SAT; BDI; STAI-state	Structured interview (diagnostic criteria)	PG:9; BE: 9; HS: 7; CS: 6; DDS: 4; punding: 8
Joutsa et al. (23)	NR	ICD+: 628 (186) ICD–: 762 (269)	NR	ICD+: 173 (80) ICD–: 216 (67)	KDDT	Diagnostic criteria	PG: 5; HS: 4; BE: 1
Leroi et al. (21)	NR	NR	NR	NR	n-back; Fluency (phonological); HADS-D; HADS-A; AES-C; BIS-11	Diagnostic criteria; SOGS	PG: 12; HS: 9; CS: 5; BE: 3; DDS: 3; punding: 3
Mack et al. (19)	NR	ICD+: 1,677.9 (893.0) ICD–: 1,269.3 (560.7)	NR	NR	Digit span; HVLT-R; TMT-A; TMT-B; Fluency (semantic, phonological); NART; BDI	Semistructured interview (diagnostic criteria)	NR
Merola et al. (42)	NR	ICD+: 1576.4 (397.6) ICD–: 1216.2 (403.0)	NR	ICD+: 344.4 (314.5) ICD–: 297.2 (235.3)	Digit span forward; Bi-syllabic words repetition test; CBTT; Paired associate learning; TMT-A; Digit cancelation test; FAB; TMT-B; MWCST; RCPM; Fluency (semantic, phonological); BDI; STAI-state; AES-C	Clinical interview (diagnostic criteria)	PG, HS, CS, punding, DDS
O'Sullivan et al. (29)	NR	ICD+: 927 (658) ICD–: 742 (477)	ICD+: 684 (512) ICD–: 588 (418)	ICD+: 259 (472) ICD–: 139 (200)	HADS-D; HADS-A; BSCS; Impulse buying tendency;	Semistructured interview (diagnostic criteria)	Punding: 20; BE: 14; HS: 12; PG: 11; CS: 11; DDS: 11
O'Sullivan et al. (28)	NR	ICD+: 981 (651) ICD–: 645 (443)	ICD+: 701 (508) ICD–: 543 (399)	ICD+: 201 (0–284)^¶^ ICD–: 0 (0–201)	HADS-D; HADS-A	Semistructured interview (diagnostic criteria)	HS: 12; PG: 11; CS: 8; BE: 8; punding: 15
Pettorruso et al. (32)	NR	PG: 712 (373) ICD+: 654 (380) ICD–: 575 (420)	PG: 592 (404) ICD+: 458 (376) ICD–: 445 (386)	PG: 120 (99) ICD+: 196 (113) ICD–: 130 (112)	FAB; HAM-D; HAM-A; SHAPS; BIS-11	Interview (diagnostic criteria)	S-ICD: 24; M-ICD: 10 (PG: 11; HS: 20; BE: 9; CS: 5)
Pineau et al. (20)	NR	ICD+: 897.5 (299.9–1247.3)^||^ ICD–: 1049.9 (527.1–1549.8)	NR	ICD+: 299.9 (77–718.0)^||^ ICD–: 340.2 (66.7–700.0)	Conner's performance test; TMT B-A; MWCST; Fluency (phonological); IGT; MADRS; Starkstein apathy scale; BIS-11	Semistructured interview; ASBPD	PG: 6; HS: 1; CS: 2; CE: 2; M-ICD: 6
Piray et al. (22)	NR	NR	NR	NR	Digit span forward; Digit span backward; Probabilistic reward learning task; NAART; BDI; BIS-11	Interview	S-ICD: 4; M-ICD: 12 (CS: 10; HS: 9; PG: 6; BE: 4)
Pontieri et al. (27)	PG: 2 ICD+: 3 ICD–:4	PG: 794 (603) ICD+: 704 (509) ICD–: 416 (304)	PG: 487 (625) ICD+: 388 (278) ICD–: 251 (279)	PG: 307 (275) ICD+: 316 (374) ICD–: 166 (197)	RAVLT (immediate, delayed); ROCF (immediate, delayed); MWCST; Stroop; Fluency (semantic, phonological); HAM-D; HAM-A; SHAPS; Starkstein apathy scale	Diagnostic criteria; QUIP	PG: 21 (PG only:10; PG and other ICD:11); HS:16; CS:3; BE:10;M-ICD: 7
Rossi et al. (10)	NR	ICD+: 935.9 (548.6) ICD–: 698.2 (474.6)	NR	ICD+: 201.9 (78.0) ICD–: 223.9 (136.8)	FAB; MWCST; Go/No-Go; Stroop; IGT; Game of dice; Investment task; Social cognition; Reversal and extinction learning; MADRS	Interview (diagnostic criteria); MIDI; SOGS;	PG: 7; HS: 2; CS: 2; DDS:2
Tessitore et al. (5)	NO	ICD+: 477.3 (222.9) ICD–: 532.1 (207.2)	NR	ICD+: 243.3 (82.1) ICD–: 243.3 (90.2)	CBTT; RAVLT (immediate, delayed); Attentional matrices; TMT-B; WCST; RCPM; Stroop; Fluency (semantic, phonological); ROCF; HAM-D; HADS	MIDI	HS:13; BE:8; PG: 1
Vela et al. (25)	NO	ICD+: 543 (248–1039)^¶^ ICD–: 460 (133–700)	ICD+: 300 (0–675)^¶^ ICD–: 300 (0–600)	ICD+: 210 (168–308)^¶^ ICD–: 180 (0–300)	BDI	QUIP	PG: 9; HS: 20; CS: 13; BE: 17; hobbyism: 25; punding: 15; walkabout: 4
Vitale et al. (6)	HS: 2 M-ICD: 0 ICD–: 0	HS: 727.3 (254.3) M-ICD: 808.3 (292.2) ICD–: 630.3 (311.8)	NR	HS: 200 (130.4) M-ICD: 207.1 (159.2) ICD–: 267.1 (201.3)	WCST; ROCF copy; TMT B-A; Attentional matrices; Stroop; RAVLT (immediate, delayed); HAM-D; HADS-A; HADS-D	MIDI; clinical interview	HS: 13; M-ICD: 10
Wu et al. (26)	NR	S-ICD: 782.3 (83.5) M-ICD: 724.0 (99.0) ICD–: 831.9 (119.2)	S-ICD: 538.0 (83.4) M-ICD: 268.5 (84.9) ICD–: 666.3 (129.0)	S-ICD: 244.3 (51.4) M-ICD: 244.0 (55.4) ICD–: 165.6 (48.9)	BDI	Semistructured interview	HS: 4; PG: 3; M-ICD: 10

*AES-C, Apathy evaluation scale by a clinician; ASBPD, Ardouin scale of behavior in Parkinson's disease; BDI, Beck depression inventory; BE, binge eating; BIS-11, Barrat impulsiveness scale-11; BSCS, Brief self-control scale CBTT, Corsi's block-tapping test; CESD, Center for Epidemiological Studies-Depression scale; CLVT-II, California verbal learning test II; CS, compulsive shopping; DA, dopamine agonist; DDS, Dopamine dysregulation syndrome; DSM-IV, diagnostic and statistical manual of mental disorders, fourth edition; DSM-IV-TR, diagnostic and statistical manual of mental disorders, fourth edition, text revision; FAB, frontal assessment battery; GDS, Geriatric depression scale; HADS-A, Hospital anxiety and depression scale—anxiety subscale; HADS-D, Hospital anxiety and depression scale—depression subscale; HAM-A, Hamilton rating scale for anxiety; HAM-D, Hamilton rating scale for depression; H and Y, Hoehn and Yahr score; HS: hypersexuality; HVLT-R, Hopkins verbal learning test revised; IA: internet addiction; ICD, impulse control disorder; ICD+, PD patients with ICD; ICD–, PD patients without ICD; IGT, Iowa gambling task; KDDT, Kirby delayed discounting questionnaire; LEDD, levodopa equivalent daily dosage (mg); LD, levodopa; MADRS, Montgomery-Asberg depression rating scale; M-ICD, multiple ICD; MIDI, Minnesota impulsive disorder interview; MWCST, Modified Wisconsin card sorting test; N, number of patients; NAART, North American adult reading test; NART, The National adult reading test; NR, not reported. PD, Parkinson's disease; PG, pathological gambling; Pts, patients; QUIP, questionnaire for impulsive-compulsive disorders in Parkinson's disease; QUIP-RS, questionnaire for impulsive-compulsive disorders in Parkinson's disease rating scale; RAVLT, Rey's auditory verbal learning test; RCPM, Raven's colored progressive matrices; Ref, reference number; ROCF, Rey-Osterrieth complex figure test; RPM, Raven's progressive matrices; SAT, salience attribution test; SHAPS, Snaith-Hamilton pleasure scale; S-ICD, single ICD; SOGS, South oaks gambling screen; STAI-state, state-trait anxiety inventory; TMT-A, trail making test part A; TMT-B, trail making test part B; UPDRS-III, unified Parkinson's disease rating scale part III (motor subscale) score; VOSP, visual object and space perception battery; WCST, Wisconsin card sorting test; WTAR, Wechsler test of adult reading; y, years*.

* *Mean (SD) unless otherwise stated*.

† *Depression as an exclusion factor*.

‡ *Data reported in months*.

§ *Mean (SEM)*.

¶ *Median (interquartile range)*.

|| *Median (lower–upper quartile)*.

** *Questionnaire or method used to screen and/or diagnose ICD*.

Four studies investigated cognitive performance without affective and motivational outcomes (8, 9, 16, 23), 17 studies included both cognitive, affective and motivational outcomes (3–6); (10, 11, 17–22); (27, 30–32, 50), and four studies included affective and motivational data only (25, 26, 28, 29). Three studies divided ICD+ in two groups: PD patients with pathological gambling and those with ICD other than pathological gambling (16, 27, 32), and one study divided the ICD+ in multiple and single ICD groups (26). As the comparison between ICD subtypes was not relevant in our meta-analysis, sub-groups were merged by calculating the pooled means and standard deviations. In one study (6) ICD+ group was divided in pathological gambling, binge-eating, hypersexuality and multiple ICD sub-groups. Since seven PD patients belonging to either the pathological gambling or the binge-eating sub-groups developed ICD before DRT initiation, only data from hypersexuality and multiple ICD sub-groups were extracted and merged as described above. Six studies focused on neuroimaging outcomes but also provided affective (26) and cognitive measures (3–5); (23, 30). One study retrospectively investigated persistent, remitting, and new-onset ICD before and after subthalamic nucleus DBS (STN-DBS) (42). For this study, only pre-STN-DBS data of persistent and never experienced ICD were included in the meta-analysis. Despite the fact that dementia was not explicitly excluded (42), data were included because STN-DBS is performed in non-demented patients only.

The meta-analysis includes 1,563 subjects. The ICD+ group was composed of 625 patients (mean age range: 54.6–68.7 years; mean PD duration: 2.4–14.3 years; mean H and Y: 1.3–2.8; mean UPDRS-III score ON medication: 10.9–36.7). The ICD– group included 938 patients (mean age: 55–71.4 years; mean PD duration: 2.3–13.1 years; mean H and Y stage: 1.4–2.5; mean UPDRS-III score ON medication: 11.7–32.3).

Fourteen meta-analyses were performed to compare cognitive outcomes and five to compare affective and motivational measures in ICD+ compared to ICD– groups.

The following cognitive outcomes were explored: short-term verbal and visuospatial memory, long-term verbal and visuospatial memory, working memory, attention, set-shifting, concept formation (reasoning, sort and shift), inhibition, cognitive flexibility, reward-related decision-making, visuospatial abilities, and language (Table 2).

**Table 2:** Cognitive subdomains and tasks used in the studies included in the meta-analysis.

**Cognitive subdomain**	**Cognitive tasks**	**References**
Short-term verbal memory	CVLT-II immediate	Erga et al. (18)
	Digit Span Forward	Biundo et al. (3, 4); Housden et al. (11); Bentivoglio et al. (17); Piray et al. (22); Merola et al. (42)
	RAVLT—immediate	Tessitore et al. (5); Vitale et al. (6); Pontieri et al. (27)
Short-term visuospatial memory	CBTT	Biundo et al. (3, 4); Tessitore et al. (5); Bentivoglio et al. (17); Merola et al. (42)
Long-term verbal memory	CVLT-II delayed HVLT-R delayed Paired associate learning Prose Memory	Erga et al. (18) Mack et al. (19) Merola et al. (42) Biundo et al. (4)
	RAVLT- delayed	Biundo et al. (3); Tessitore et al. (5); Vitale et al. (6); Bentivoglio et al. (17); Pontieri et al. (27)
Long-term visuospatial memory	ROCF—delayed	Biundo et al. (3, 4); Pontieri et al. (27)
Working memory	Digit Ordering Test	Biundo et al. (4)
	Digit Span Backward	Biundo et al. (3); Djamshidian et al. (8); Housden et al. (11); Bentivoglio et al. (17); Piray et al. (22)
	n-Back	Leroi et al. (21)
Attention	Attentive Matrices	Tessitore et al. (5); Vitale et al. (6)
	Conner's Performance Test	**Pineau et al. (20)**
	Double barrage—accuracy	Bentivoglio et al. (17)
	TMT-A	**Biundo et al. (3, 4); Mack et al. (19); Merola et al. (42)**
Set-shifting	TMT-B	**Biundo et al. (3, 4); Tessitore et al. (5); Mack et al. (19); Merola et al. (42)**
	TMT- B-A	**Vitale et al. (6); Pineau et al. (20)**
Concept formation (sort and shift)	MWCST—categories	Rossi et al. (10); Bentivoglio et al. (17); Pineau et al. (20)**;** Pontieri et al. (27); Merola et al. (42)
	WCST—global score	**Vitale et al. (6); Tessitore et al. (5)**
Concept formation (reasoning)	RCPM RPM	Biundo et al. (3); Tessitore et al. (5); Bentivoglio et al. (17); Merola et al. (42) Cilia et al. (30)
Inhibition	Go/no-Go—errors	**Rossi et al. (10)**
	Stop Signal Task	Claassen et al. (31)
	Stroop errors	**Biundo et al. (3, 4); Vitale et al. (6);Djamshidian et al. (9); Bentivoglio et al. (17)**
	Stroop time	**Tessitore et al. (5); Cera et al. (16); Erga et al. (18); Pontieri et al. (27)**
Cognitive flexibility	Phonological Fluency	Biundo et al. (3, 4); Tessitore et al. (5); Bentivoglio et al. (17); Erga et al. (18); Mack et al. (19); Leroi et al. (21); Pineau et al. (20); Pontieri et al. (27); Merola et al. (42)
Reward-related decision-making	IGT	**Rossi et al. (10); Bentivoglio et al. (17); Pineau et al. (20)**
	KDDQ	Housden et al. (11); Joutsa et al. (23)
	Monetary risk taking	Cera et al. (16)
	Probabilistic Reward	Piray et al. (22)
	Risk Task	Djamshidian et al. (8)
Visuospatial abilities	Constructional apraxia	Bentivoglio et al. (17)
	ROCF—copy	Biundo et al. (3, 4); Tessitore et al. (5); Vitale et al. (6); Pontieri et al. (27)
	VOSP—silhuette	Erga et al. (18)
Language	Naming	Biundo et al. (4)
	Oral Verbal Naming	Bentivoglio et al. (17)
**Affective and Motivational**	**Self-report measures**	**References**
Depression	BDI	Biundo et al. (3, 4); Housden et al. (11); Mack et al. (19); Piray et al. (22); Vela et al. (25); Wu et al. (26); Merola et al. (42)
	CESD	Claassen et al. (31)
	GDS	Cilia et al. (30)
	HADS-D	Vitale et al. (6); Leroi et al. (21); O'Sullivan et al. (28, 29)
	HAM-D	Tessitore et al. (5); Bentivoglio et al. (17); Pontieri et al. (27); Pettorruso et al. (32)
	MADRS	Rossi et al. (10); Erga et al. (18); Pineau et al. (20)
Anxiety	HADS-A	Tessitore et al. (5); Vitale et al. (6); Leroi et al. (21); O'Sullivan et al. (28, 29)
	HAM-A	Bentivoglio et al. (17); Pontieri et al. (27); Pettorruso et al. (32)
	STAI-state	Housden et al. (11); Merola et al. (42)
Anhedonia	SHAPS	Pontieri et al. (27); Pettorruso et al. (32)
Apathy	AES-C	Leroi et al. (21); Merola et al. (42)
	Starkstein Apathy Scale	Pineau et al. (20); Pontieri et al. (27)
Impulsivity	BIS-11	Bentivoglio et al. (17); Pineau et al. (20); Leroi et al. (21); Piray et al. (22); Pettorruso et al. (32)
	BSCS	O'Sullivan et al. (29)

*AES-C, Apathy evaluation scale by a clinician; BDI, Beck depression inventory; BIS-11, Barrat impulsiveness scale-11; BSCS, brief self-control scale; CBTT, Corsi's block-tapping test; CVLT-II, California verbal learning test II; CESD, Centre for Epidemiological Studies-Depression scale; GDS, Geriatric depression scale; HADS-A, Hospital anxiety and depression scale-anxiety subscale; HADS-D, Hospital anxiety and depression scale-depression subscale; HAM-A, Hamilton rating scale for anxiety; HAM-D, Hamilton rating scale for depression; HVLT-R, Hopkins verbal learning test revised; IGT, Iowa gambling task; KDDQ, Kirby delayed discounting questionnaire; MADRS, Montgomery-Asberg depression rating scale; MWCST, modified Wisconsin card sorting test; RAVLT, Rey's auditory verbal learning test; RCPM, Raven's colored progressive matrices; ROCF, Rey-Osterrieth complex figure test; RPM, Raven's progressive matrices; SHAPS, Snaith-Hamilton pleasure scale; STAI-state, state-trait anxiety inventory; TMT-A, trail making test part A; TMT-B, trail making test part B; VOSP, visual object and space perception battery; WCST, Wisconsin card sorting test. In bold scores that have been reversed in order to obtain scores with the same meaning (e.g., higher scores better performances)*.

**Figure 2:**
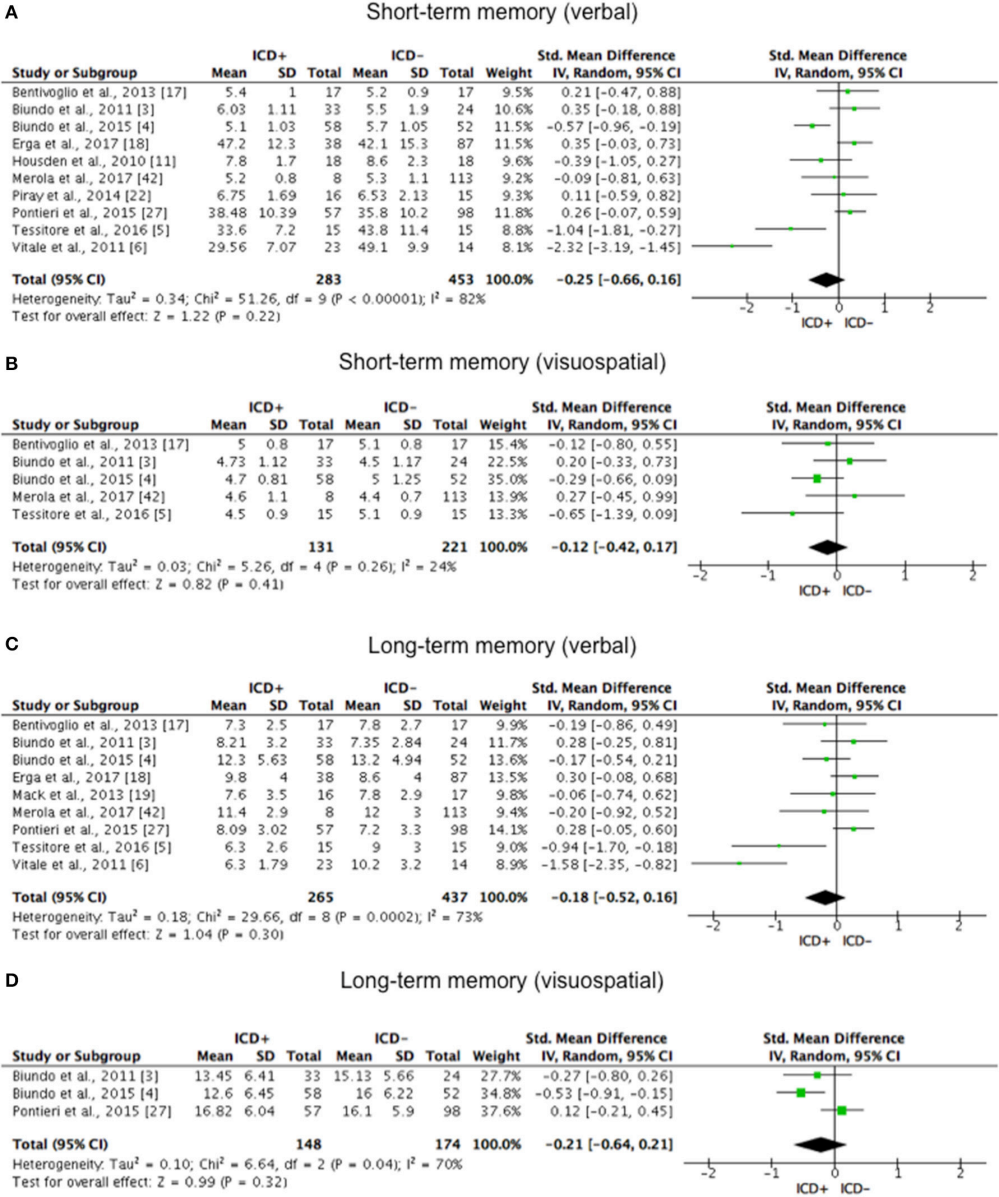
Forest plots for memory. Here are reported forest plots for short-term (verbal, **(A)** visuospatial, **(B)** and long-term (verbal, **(C)**; visuospatial, **(D)** memory outcomes. Standardized mean difference represents Hedges's g effect size. The size of the square indicates the weight of the study. The horizontal line represents the 95% confidence interval. The diamond represents the pooled effect size. Negative effect sizes indicate worse performance in PD patients with ICD (ICD+) in comparison to those without ICD (ICD–). ICD, impulse control disorder; PD, Parkinson's disease.

**Figure 3:**
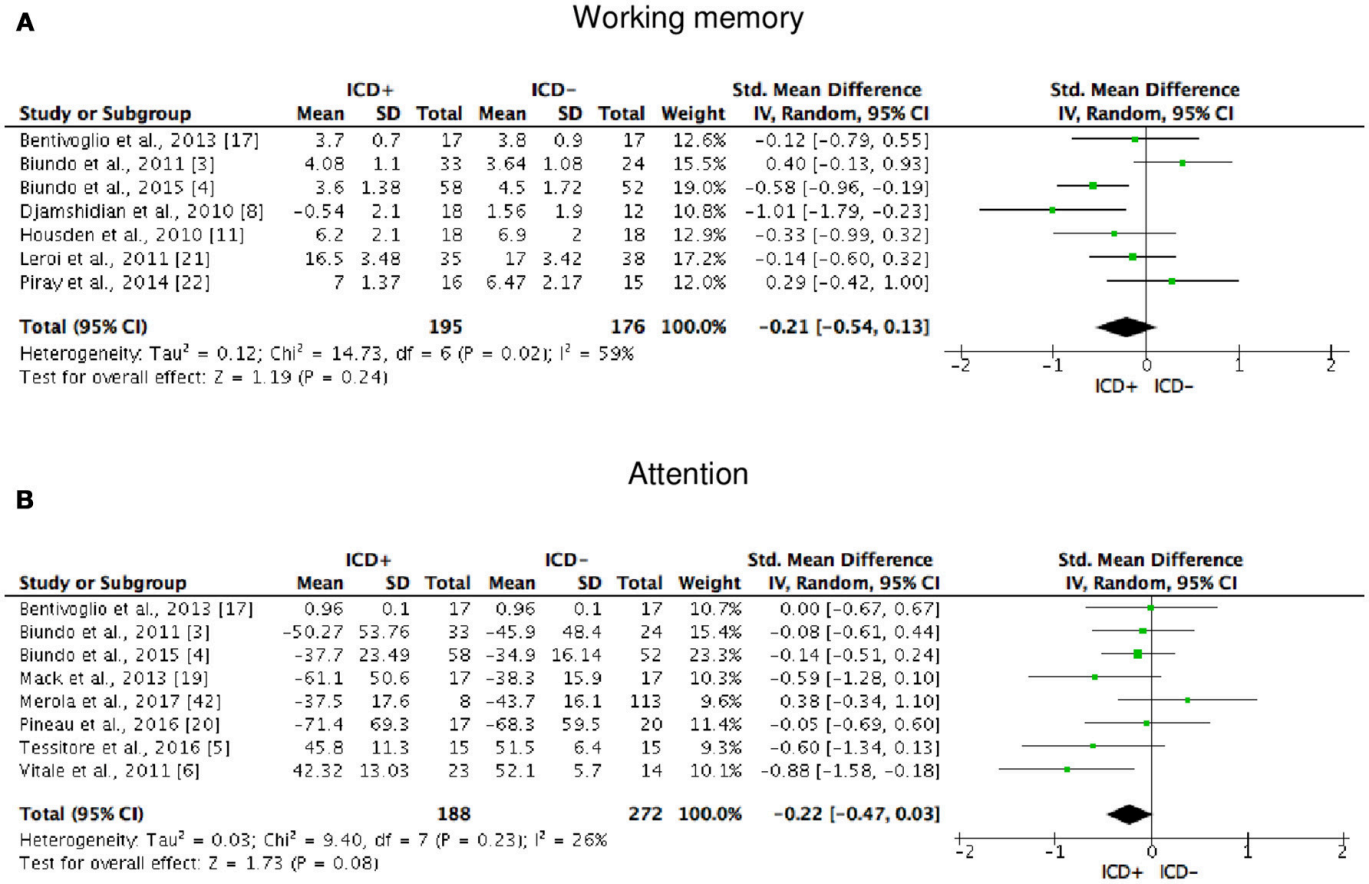
Forest plots for working memory and attention. Here are reported forest plots for working memory **(A)** and attention **(B)**. Standardized mean difference represents Hedges's g effect size. The size of the square indicates the weight of the study. The horizontal line represents the 95% confidence interval. The diamond represents the pooled effect size. Negative effect sizes indicate worse performance in PD patients with ICD (ICD+) in comparison to those without ICD (ICD−). ICD, impulse control disorder; PD, Parkinson's disease.

**Figure 4:**
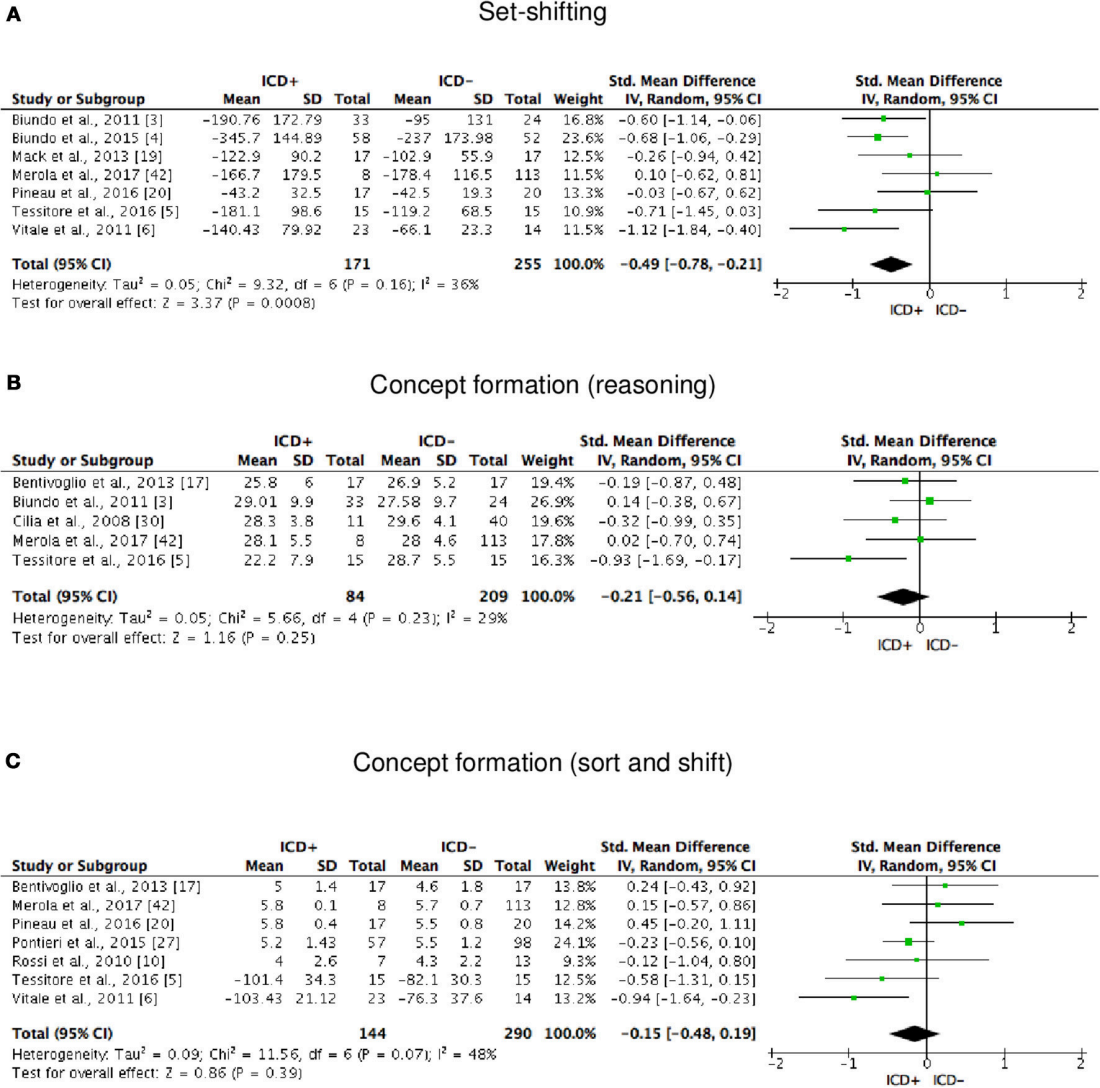
Forest plots for executive functions set-shifting and concept formation. Here are reported forest plots for set-shifting **(A)**, and concept formation (reasoning, **B**; sort and shift, **C**).

**Figure 5:**
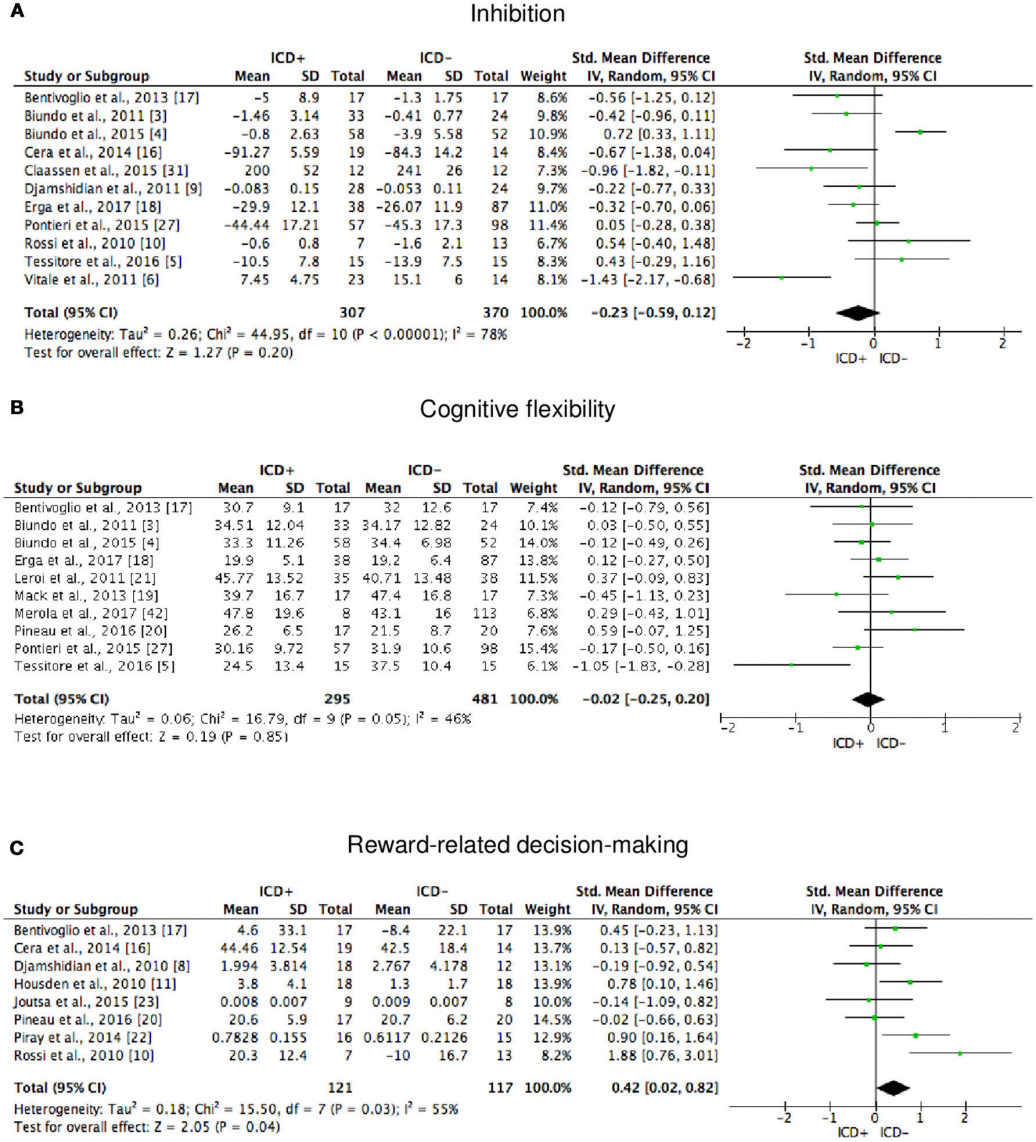
Forest plots for executive functions inhibition, cognitive flexibility, and reward-related decision-making. Here are reported forest plots for inhibition **(A)**, cognitive flexibility **(B)**, and reward-related decision-making **(C)**. Standardized mean difference represents Hedges's g effect size. The size of the square indicates the weight of the study. The horizontal line represents the 95% confidence interval. The diamond represents the pooled effect size. Negative effect sizes indicate worse performance in PD patients with ICD (ICD+) in comparison to those without ICD (ICD–). ICD, impulse control disorder; PD, Parkinson's disease.

**Figure 6:**
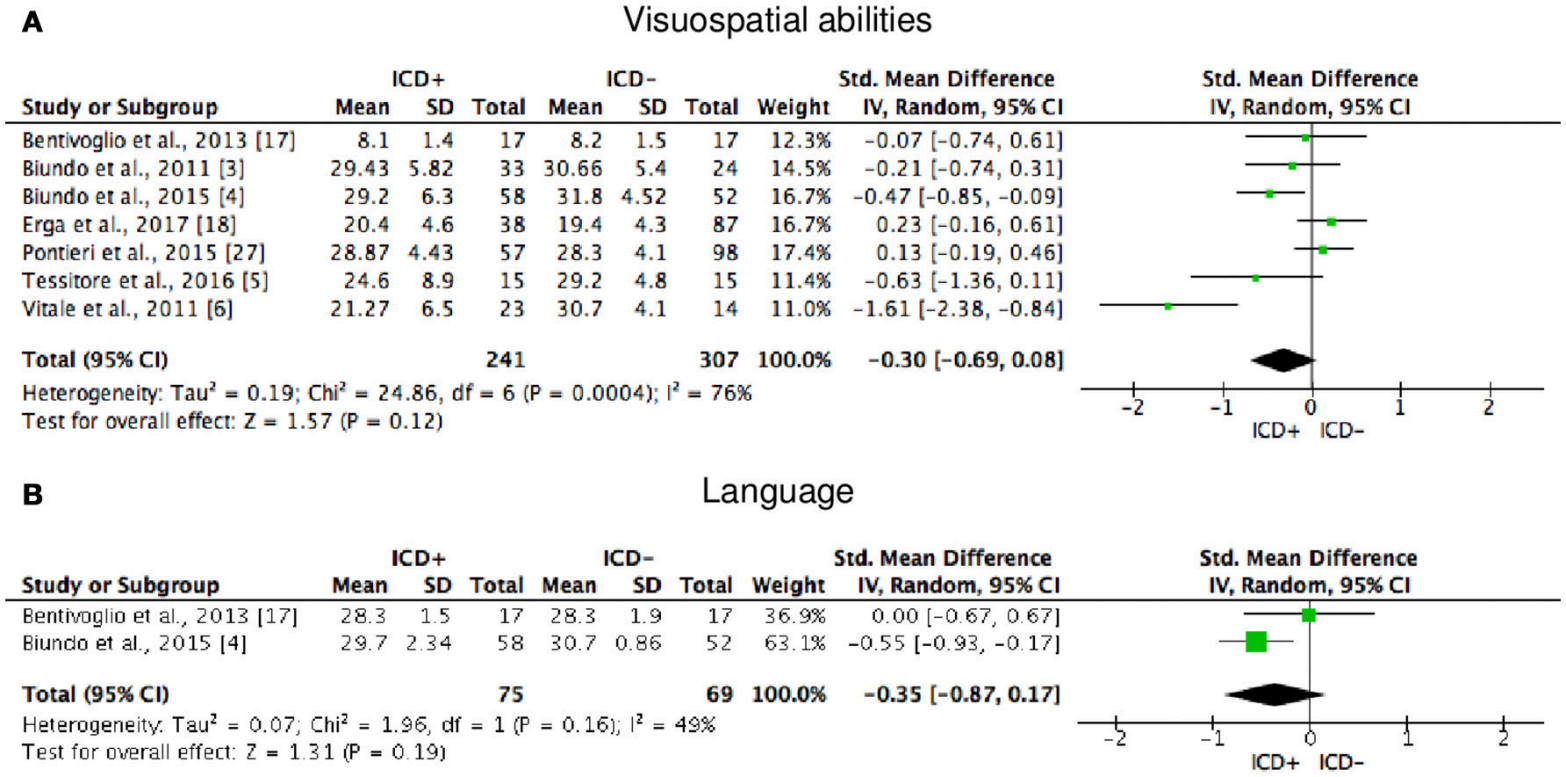
Forest plots for visuospatial abilities and language. Here are reported forest plots for visuospatial abilities **(A)** and language **(B)**. Standardized mean difference represents Hedges's g effect size. The size of the square indicates the weight of the study. The horizontal line represents the 95% confidence interval. The diamond represents the pooled effect size. Negative effect sizes indicate worse performance in PD patients with ICD (ICD+) in comparison to those without ICD (ICD−). ICD, impulse control disorder; PD, Parkinson's disease.

ICD+ showed worse performance in set-shifting (SMD = −0.49; 95% CI: −0.78, −0.21; Z = 3.37; *p* = 0.0008) and reward-related decision-making (SMD = 0.42; 95% CI: 0.02, 0.82; Z = 2.05; *p* = 0.04). The heterogeneity was low-to-moderate for set-shifting (χ^2^ = 9.32, *p* = 0.16, *I*^2^ = 36%) and moderate for reward-related decision-making (χ^2^ = 15.50, *p* = 0.03, *I*^2^ = 55%). Effect sizes for the other cognitive outcomes did not differ significantly between groups. Heterogeneity was low for short-term visuospatial memory, attention, concept formation (reasoning), moderate for cognitive flexibility, concept formation (sort and shift), and language, high for short-term verbal memory, long-term verbal memory, long-term visuospatial memory, visuospatial abilities, and inhibition, moderate-to-high for working memory (Figures 2–6).

**Figure 7:**
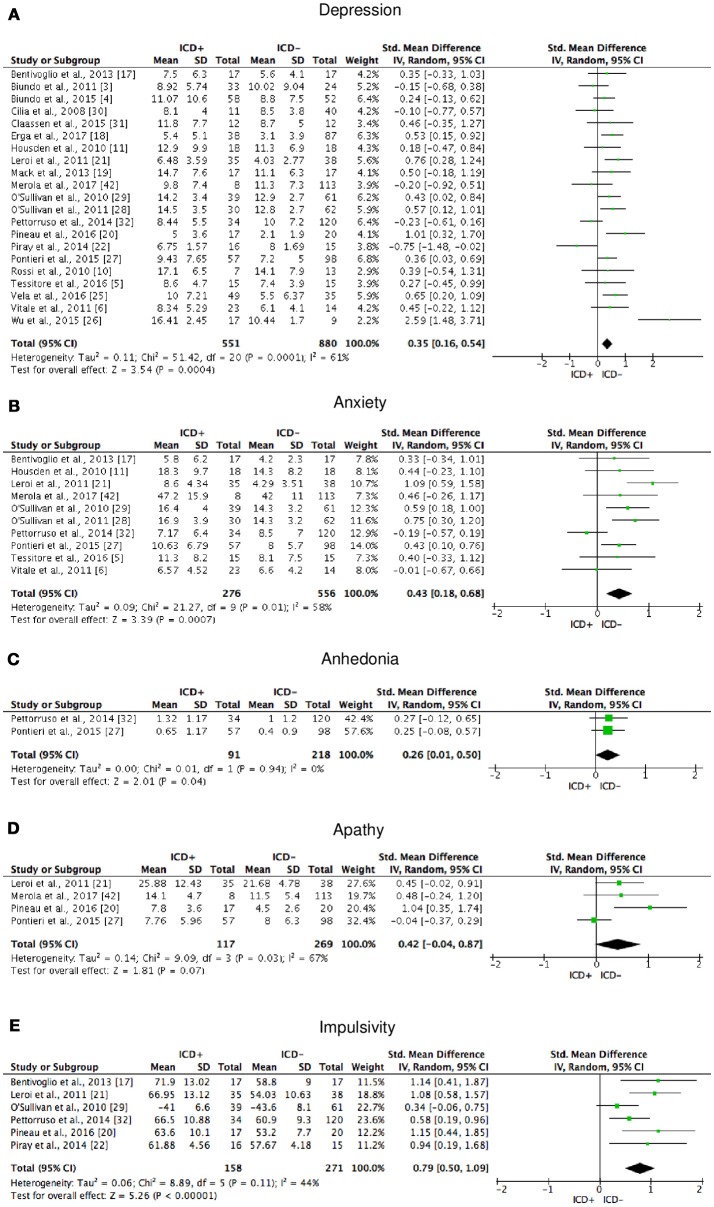
Forest plots for affective and motivational outcomes. Here are reported forest plots for depression **(A)**, anxiety **(B)**, anhedonia **(C)**, apathy **(D)**, and impulsivity **(E)**. Standardized mean difference represents Hedges's g effect size. The size of the square indicates the weight of the study. The horizontal line represents the 95% confidence interval. The diamond represents the pooled effect size. Negative effect sizes indicate worse performance in PD patients with ICD (ICD+) in comparison to those without ICD (ICD−). ICD, impulse control disorder; PD, Parkinson's disease.

The following self-reported affective and behavior outcomes were explored: depression, anxiety, anhedonia, apathy, and impulsivity. ICD+ showed increased depression (SMD = 0.35; 95% CI: 0.16, 0.54; Z = 3.54; *p* = 0.0004), anxiety (SMD = 0.43; 95% CI: 0.18, 0.68; Z = 3.39; *p* = 0.0007), anhedonia (SMD = 0.26; 95% CI: 0.01, 0.50; Z = 2.01; *p* = 0.04), and impulsivity (SMD = 0.79; 95% CI: 0.50, 1.09; Z = 5.26; *p* < 0.00001), but comparable apathy symptoms (Figure 7). Heterogeneity was low for anhedonia (χ^2^ = 0.01, *p* = 0.94, *I*^2^ = 0%), moderate for impulsivity (χ^2^ = 8.89, *p* = 0.11, *I*^2^ = 44%), and moderate-to-high for depression (χ^2^ = 51.42, *p* = 0.0001, *I*^2^ = 61%), anxiety (χ^2^ = 21.27, *p* = 0.01, *I*^2^ = 58%), and apathy (χ^2^ = 9.09, *p* = 0.03, *I*^2^ = 67%; Figure 7). Results of the meta-analyses are summarized in Table 3.

**Table 3:** Results of the meta-analyses.

			**Random-effect model results**				**Heterogeneity**		
**Outcome**	**K**	**N**	**SMD**	**[95% CI]**	**Z**	***p***	***X^2^***	***p***	***I^2^*(%*)***
Short-term verbal memory	10	736	−0.25	[−0.66, 0.16]	1.22	0.22	51.26	<*0.00001*	82
Short-term visuospatial memory	5	352	−0.12	[−0.42, 0.17]	0.82	0.41	5.26	0.26	24
Long-term verbal memory	9	702	−0.18	[−0.52, 0.16]	1.04	0.30	29.66	*0.0002*	73
Long-term visuospatial memory	3	322	−0.21	[−0.64, 0.21]	0.99	0.32	6.64	*0.04*	70
Working memory	7	371	−0.21	[−0.54, 0.13]	1.19	0.24	14.73	*0.02*	59
Attention	8	460	−0.22	[−0.47, 0.03]	1.73	0.08	9.40	0.23	26
Set-shifting	7	426	−0.49	[−0.78, −0.21]	3.37	*0.0008*	9.32	0.16	36
Concept formation (sort and shift)	7	434	−0.15	[−0.48, 0.19]	0.86	0.39	11.56	0.07	48
Concept formation (reasoning)	5	293	−0.21	[−0.56, 0.14]	1.16	0.25	5.66	0.23	29
Inhibition	11	677	−0.23	[−0.59, 0.12]	1.27	0.20	44.95	<*0.00001*	78
Cognitive flexibility	10	776	−0.02	[−0.25, 0.20]	0.19	0.85	16.79	*0.05*	46
Reward-related decision-making	8	238	0.42	[0.02, 0.82]	2.05	*0.04*	15.50	*0.03*	55
Visuospatial abilities	7	548	−0.30	[−0.69, 0.08]	1.57	0.12	24.86	*0.0004*	76
Language	2	144	−0.35	[−0.87, 0.17]	1.31	0.19	1.96	0.16	49
Depression	21	1431	0.35	[0.16, 0.54]	3.54	*0.0004*	51.42	*0.0001*	61
Anxiety	10	832	0.43	[0.18, 0.68]	3.39	*0.0007*	21.27	*0.01*	58
Anhedonia	2	309	0.26	[0.01, 0.50]	2.01	*0.04*	0.01	0.94	0
Apathy	4	386	0.42	[−0.04, 0.87]	1.81	0.07	9.09	*0.03*	67
Impulsivity	6	429	0.79	[0.50, 1.09]	5.26	<*0.00001*	8.89	0.11	44

*K, number of studies; N, number of participants; SMD, standardized mean difference; CI, confidence interval. P values below the significance level (p < 0.05) are reported in italics*.

### Risk of bias

**Figure 8:**
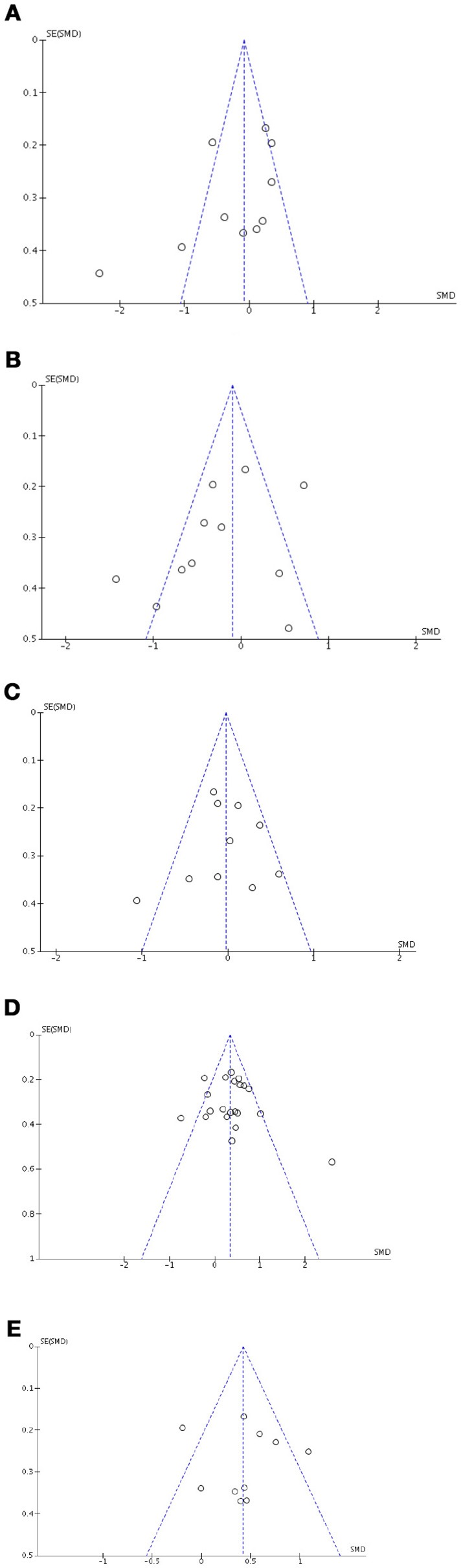
Funnel plots for cognitive, affective and motivational outcomes. Here are reported funnel plots for short-term verbal memory **(A)**, inhibition **(B)**, phonological fluency **(C)**, depression **(D)**, and anxiety **(E)**. There is no evidence to suggest publication bias.

Visual exploration of funnel plots did not suggest possible publication bias for short-term verbal memory, inhibition, cognitive flexibility, depression, and anxiety that were the only outcomes with at least 10 studies (Figure 8).

Risk of performance bias was unclear with only 2/25 studies indicating assessors blinding procedures.

Attrition bias was low, with 4/25 studies with missing data.

### Sensitivity analysis and moderator analysis

Sensitivity analysis showed that after removing Pontieri et al. (27), the overall effect size of long-term visuospatial memory became significant (SMD = −0.44; 95% CI: −0.75, −0.13; Z = 2.81; *p* = 0.005) and the heterogeneity changed from high (χ^2^ = 6.64, *p* = 0.04, *I*^2^ = 70%) to low (χ^2^ = 0.62, *p* = 0.43, *I*^2^ = 0%). After removing Biundo et al. (3), the overall effect size of working memory became significant (SMD = −0.32; 95% CI: −0.63, −0.01; Z = 2.05; *p* = 0.04) and the heterogeneity changed from high (χ^2^ = 14.73, *p* = 0.02, *I*^2^ = 59%) to moderate (χ^2^ = 8.41, *p* = 0.13, *I*^2^ = 41%). The overall effect size of attention became significant after removing Merola et al. (42) (SMD = −0.27; 95% CI: −0.50, −0.04; Z = 2.29; *p* = 0.02), but heterogeneity remained low. The overall effect size of inhibition became significant after removing Biundo et al. (4) (SMD = −0.34; 95% CI: −0.65, −0.03; Z = 2.18; *p* = 0.03) and heterogeneity changed from high to moderate-to-high (χ^2^ = 24.18, *p* = 0.004, *I*^2^ = 63%). The overall effect size of reward-related decision-making lost significance after removing Bentivoglio et al. (17) (SMD = 0.42; 95% CI: −0.05, 0.89; Z = 1.75; *p* = 0.08), Housden et al. (11) (SMD = 0.36; 95% CI: −0.08, 0.81; Z = 1.59; *p* = 0.11), Piray et al. (22) (SMD = 0.35; 95% CI: −0.08, 0.78; Z = 1.58; *p* = 0.11), and Rossi et al. (10) (SMD = 0.29; 95% CI: −0.03, 0.61; Z = 1.78; *p* = 0.07). After removing Rossi et al. (10), heterogeneity changed from moderate (χ^2^ = 15.50, *p* = 0.03, *I*^2^ = 55%) to low (χ^2^ = 8.27, *p* = 0.22, *I*^2^ = 27%). Including or excluding the other studies did not change heterogeneity. The overall effect size of apathy became significant after removing Pontieri et al. (27) (SMD = 0.60; 95% CI: 0.25, 0.95; Z = 3.38; *p* = 0.0007) and heterogeneity changed from high (χ^2^ = 9.09, *p* = 0.03, *I*^2^ = 67%) to low (χ^2^ = 2.07, *p* = 0.35, *I*^2^ = 4%). Moderator analysis was performed for short-term verbal memory, inhibition, cognitive flexibility, and depression, which were the only outcomes that included at least 10 studies each (51). Anxiety did not undergo moderator analysis, because none of the covariates of interest were assessed in at least 10 studies. Moderator analysis showed no effect of age, education, PD duration, H and Y, UPDRS-III, and total LEDD, levodopa LEDD, dopamine agonist LEDD on short-term verbal memory, inhibition, cognitive flexibility, and depression (Table 4).

**Table 4:** Results of the moderator analysis.

	**Short-term verbal memory**			**Inhibition**			**Cognitive flexibility**			**Depression**			**Anxiety**		
**Moderators**	**K**	**β**	***p***	**K**	**β**	***p***	**K**	**β**	***p***	**K**	**β**	***p***	**K**	**β**	***p***
Age	9^a^	–	–	11	−0.003	0.970	8^a^	–	–	19	−0.029	0.183	8^a^	–	–
Education	8^a^	–	–	10	−0.050	0.669	6^a^	–	–	10	−0.055	0.332	6^a^	–	–
PD Duration	8^a^	–	–	10	0.045	0.645	9^a^	–	–	19	−.012	0.810	8^a^	–	–
H and Y	8^a^	–	–	8^a^	–	–	6^a^	–	–	14	−0.153	0.570	7^a^	–	–
UPDRS–III	10	0.073	0.081	11	0.018	0.578	10	−0.005	0.799	19	−0.009	0.557	9^a^	–	–
Total LEDD	9^a^	–	–	10	0.002	0.200	9^a^	–	–	19	0.000	0.992	9^a^	–	–
DA LEDD	9^a^	–	–	9^a^	–	–	8^a^	–	–	18	0.001	0.435	9^a^	–	–
LD LEDD	4^a^	–	–	5^a^	–	–	3^a^	–	–	10	0.000	0.749	6^a^	–	–

*PD, Parkinson's disease; H and Y, Hoehn and Yahr score; UPDRS–III, unified Parkinson's disease rating scale part III (motor subscale) score; LEDD, levodopa equivalent daily dosage (mg); DA, dopamine agonist; LD, levodopa; K, number of studies*.

a *not included in the moderator analysis because k < 10*.

## Discussion

The primary aim of this meta-analysis of 25 studies was to describe the pattern of cognitive function in DRT-medicated ICD+ compared to ICD–. A stricter set of inclusion criteria was applied than used previously (33), to achieve a more homogenous ICD+ group, and a better understanding of the relationship between ICD and cognition in medicated PD. A secondary aim was to examine affective and motivational correlates of ICD, as emotion-cognition and motivation-cognition relationships are receiving increasing attention to understand psychopathology and improve pharmacological and psychological treatments (34).

Our findings suggest ICD to be associated with worse performance on a set of executive function measures assessing set-shifting (Trail Making Test part B, and B-A) and reward-related decision-making (Iowa Gambling Task, Monetary Risk Task, Kirby Delay Discounting Questionnaire), with relative sparing of other executive tasks that assess concept formation and reasoning (Raven's progressive matrices standard and colored versions), concept formation sort and shift (Wisconsin card sorting test standard and modified versions), inhibition (Stroop, Stop Signal Task, Go/no-Go), and cognitive flexibility (phonological fluency), as well as memory, working memory, attention, visuospatial abilities, and language.

Set-shifting and reward-related decision-making abilities are important determinants of advantageous behavior, serving to translate goals into action planning, as well as monitoring response and errors (52).

Structural and functional neuroimaging outcomes were not included in this meta-analysis, but neuroanatomical findings in patients with abnormalities in set-shifting and reward-related decision-making may help speculate on brain areas that may undergo DRT overdose in PD. Lesion-symptom mapping studies suggest reward-related decision-making to rely upon an anatomical network composed of the ventromedial, orbitofrontal and frontopolar cortices. Set-shifting, which is one of the processes underlying cognitive control, depends on rostral anterior cingulate cortex functioning (52). These brain areas form part of the mesocorticolimbic system that, in the early stages of PD, undergo less dopaminergic damage than the dorsal striatal pathways.

According to the “overdose hypothesis,” the DRT amount required to control motor symptoms in PD has the potential to move the same patient away from the optimum for certain cognitive functions (53). The relationship between the efficiency of neuronal activity and the state of dopaminergic modulation is represented by a Yerkes-Dodson inverted U-shaped curve with cognitive functions declining with deviation away from optimum dopamine levels, indicated by the center of the curve (2). Extrapolating this model to set-shifting and reward-related decision-making implies that DRT has the capacity to both improve and impair these executive functions depending on baseline dopamine levels in the underlying neural circuitry. For patients with low baseline dopamine levels in the mesocorticolimbic system, DRT may optimize activity as supported by improved set-shifting and reward-related decision-making when assessed in an optimally medicated state compared to the same patients assessed following DRT withdrawal (54, 55). By the same token, if patients start out with higher mesocorticolimbic baseline levels of dopamine, DRT causes dopamine over-activity in the mesocorticolimbic system. This view is consistent with evidence that dopamine agonists increase frontal cortex blood flow (56), and enhance reward-related risk-taking behavior in ICD+ compared to ICD– (57).

A recent meta-analysis of case-control studies on the prevalence of ICD in PD provides indirect evidence of dopaminergic over-activity, as being medicated for PD and disease duration were both factors that increased the risk of ICD (58). As disease duration advances, the dopaminergic degeneration spread to brain areas that were spared in the early stages of the disease, such as prefrontal cortex (59). The progressive involvement of brain areas during PD progression may have two consequences. The first is a dysregulation of brain regions involved in the top-down mechanisms of cognitive control of behavior (60). The second is the need to increase DRT dosage to compensate motor symptoms and the consequent overstimulation of less damaged brain areas. However, the relationship between ICD and DRT dosage is not well-established; some studies report no difference between DRT doses and ICD (18, 25, 61, 62), with others reporting an association between ICD and dopamine agonists doses (63–68). In this meta-analysis we lacked the power for conducting moderator analysis for disease duration, total LEDD, LD LEDD, and DA LEDD in reward-related decision-making and set-shifting leaving this question unanswered.

Our data may help reconcile the debate whether ICD in PD is associated with frontal lobe dysfunction (69–72). The discrepancy between previous reports is likely due to differences in the tasks and the underlying executive function subdomains investigated. Our data indicate that some frontal tasks and related subdomains may not be affected by ICD. Therefore, neuropsychological evaluation of ICD+ patients should include a broad range of executive function tasks, encompassing both reward-related decision-making and set-shifting, and not be limited to a general frontal screening test, such as the Frontal Assessment Battery, which does not include those subdomains.

The profile of executive dysfunction we found confirms the conclusions of a previous meta-analysis (33) that also reported reduced abstraction/concept formation and visuospatial abilities in ICD+. The discrepancy between the two meta-analyses can be ascribed to our inclusion of two reports (18, 50) not available at the time of the former one, and by our stricter exclusion criteria. We excluded four studies included by Santangelo et al. (7, 14, 58, 59), because of (a) patients with hypersexuality and compulsive shopping included the ICD– group (7), (b) dementia not excluded (14), and (c) patients screened for pathological gambling (73) or punding (74) only, thereby the presence of other ICDs in the ICD– group could not be ruled out.

Our secondary aim was to explore affective and motivational outcomes associated with ICD, as evidence indicates a role for dopamine dysregulation in the pathophysiology of impulsivity, apathy, and anhedonia in pathological gambling, drug addiction, and ICD+ (75–77). We found increased rates of self-reported depression, anxiety, anhedonia, and impulsivity, but not apathy in ICD+ compared to ICD–.

Impulsivity and apathy have been suggested to represent opposite ends of a dopaminergic continuum, where the former and the latter are associated with hyper and hypodopaminergic state, respectively (75). According to this view, DRT mesocorticolimbic overstimulation increases impulsivity that, in turn, may enhance reward-related behavior that, over time, may become addictive in nature (78). The association between ICD+ and impulsivity but not apathy in our meta-analysis is consistent with this model and the evidence that the D2 dopamine agonist pramipexole improves apathy in PD patients without ICD (79) but also increases impulsivity (1).

Anhedonia is defined as the decreased ability to experience pleasure from positive stimuli (80). Pramipexole may reduce anhedonia in ICD–, suggesting its hypodopaminergic nature (81).

The co-occurrence of hypodopaminergic anhedonia with hyperdopaminergic ICD is surprising. One possible explanation is that ICD+ patients may have decreased ability to experience pleasure when not engaged in ICD. This hypothesis is supported by the evidence that people addicted to alcohol or drugs experience anhedonia during withdrawal syndrome, a feature that may facilitate relapse (82). However, the relationship between anhedonia and dopaminergic states is not so straightforward and anhedonia is also recognized as one of the overlapping symptoms between apathy and depression (83). The association with anhedonia may be confounded by the presence of depression, which in some cases might be serotoninergically mediated (84). However, there are only two studies and further investigation is needed.

The pathophysiology of depression and anxiety in PD is likely to be multifactorial including reaction to disease diagnosis and anxiety about its future course. Depression and anxiety are present in the premorbid PD stage (85), therefore suggesting they may represent a core feature of PD. In our meta-analysis depression and anxiety levels were higher in ICD+ compared to ICD–. ICD may have a negative impact on the quality of life (21, 25), and in turn increase depression and anxiety levels. Also, as the mesocorticolimbic pathways dysfunction may be involved in depression, anxiety and ICD, they might co-occur as epiphenomena of shared neural correlates (40).

The main limitation of this meta-analysis is the small number of studies, most of which with small samples that might have contributed to high heterogeneity for some of the outcomes explored. This consideration could be reflected in the sensitivity analysis data for long-term visuospatial memory, working memory, attention, inhibition, reward-related decision-making, apathy, and it suggests caution in the interpretation of the results for these outcomes. Moreover, the inclusion in the same domains of tasks that might involve different cognitive processes could have contributed to the high heterogeneity and the low stability of some results. However, considering the single cognitive task would have resulted in a reduction of the power, because of the low number of studies using the same tasks. Unfortunately, we were not able to perform separate analyses for dopamine agonists and levodopa, as the majority of the studies included patients who were under both types of DRT. Due to the small number of studies, moderator analysis for levodopa and dopamine agonist LEDD was performed for depression only, which showed no effect. This is not surprising, as in the larger study published so far, ICDs were found to be associated either with dopamine agonists or, to a lesser extent, with levodopa (1). These data are in keeping with the notion that both levodopa and dopamine agonists can interfere with the phasic and tonic activity of dopaminergic neurons (86) that, by facilitating neuroadaptive changes in dopaminergic system functioning, may predispose to ICD.

Another limitation is the inclusion of cross-sectional studies that impede the exploration of the direction of the cause-effect relationship between cognitive, affective and motivational outcomes and ICD; therefore multi-center and longitudinal studies are needed. Moreover, even if we excluded studies focusing on punding and dopamine dysregulation syndrome only, these conditions were present in many studies, and probably contributed to high heterogeneity for some outcomes. Furthermore, 23/25 studies did not mention assessors to be blind to the ICD status and this might have affected tools administration and scoring. Future studies should be conducted following blinding procedures. Finally, QUIP, a validated screening instrument with high sensitivity (94%) but low specificity (72%) to ICD in PD (87) was used in two studies (18, 25), possibly leading to false positive and/or subclinical ICD inclusion. Still unanswered questions include whether set-shifting and reward-related decision-making abnormalities in PD patients with ICD reflect structural and functional mesocorticolimbic changes due to acute or chronic DRT effects, or whether they can revert following ICD treatment and remission. Future studies should address these points, since better understanding ICD pathophysiology may help tailoring treatment of ICD+.

## Author contributions

The study has been designed by AM, DD, NE, and ST. Data have been gathered and analyzed by AM and DD under the supervision of JG. The manuscript has been drafted by AM, NE, and ST. AM, DD, NE, JG, and ST revised the manuscript.

### Conflict of interest statement

The authors declare that the research was conducted in the absence of any commercial or financial relationships that could be construed as a potential conflict of interest.

## Abbreviations

DBS: deep brain stimulation
DRT: dopamine replacement treatment
H and Y: Hoehn and Yahr scale
ICD: impulse control disorder
LEDD: levodopa equivalent daily dose
PD: Parkinson's disease
QUIP: Questionnaire for Impulsive-Compulsive Disorders in Parkinson's Disease
SDM: standardized mean difference
STN-DBS: sub thalamic nucleus deep brain stimulation
UPRDS: Unified Parkinson's Disease Rating Scale.
